# The Absence of CXCL10 Activity Does Not Affect the Capability of CD8^+^ T Cells to Migrate and Eliminate the Tissue Cysts of *Toxoplasma gondii* from the Brains of Chronically Infected Mice

**DOI:** 10.3390/microorganisms12112172

**Published:** 2024-10-29

**Authors:** Rajesh Mani, Yasuhiro Suzuki

**Affiliations:** Department of Microbiology, Immunology and Molecular Genetics, University of Kentucky College of Medicine, Lexington, KY 40536, USA

**Keywords:** *Toxoplasma gondii*, cyst, protective immunity, CD8^+^ T cell, CXCL10

## Abstract

*Toxoplasma gondii* forms tissue cysts in neurons and astrocytes in the brain to establish chronic infection, and astrocytes express the CXCL10 chemokine in chronically infected mice. Since chemokines mediate the migration of T cells to attack their targets, and since CXCL10 plays key roles in T cell-mediated control of the proliferation of tachyzoites (the acute stage form) of *T. gondii* during the acute stage of infection, we examined whether CXCL10 is involved in recruiting anti-cyst CD8^+^ cytotoxic T cells to eliminate the cysts in their brains. We employed adoptive transfer of CD8^+^ immune T cells to infected, T cell-deficient SCID and RAG1^−/−^ mice in combination with blocking CXCL10 activity by neutralizing antibody or a deletion of this chemokine gene. The treatment of chronically infected (infected and treated with sulfadiazine) SCID mice with the anti-CXCL10 antibody did not inhibit the recruitment of the transferred CD8^+^ T cells into their brains and the removal of cerebral *T. gondii* cysts by the T cells. In addition, the neutralization of CXCL10 did not reduce the cerebral expression of mRNA for the mediators (perforin and granzyme B [GzmB]) of the cytotoxic activity of CD8^+^ T cells in the SCID mice. Consistently, the adoptive transfer of CD8^+^ immune T cells to chronically infected RAG1^−/−^CXCL10^−/−^ mice did not show any defects in recruiting the CD8^+^ T cells into their brains and eliminating the cysts when compared to infected RAG1^−/−^ mice. The former rather displayed enhanced cyst removal with increased cerebral expression of GzmB mRNA. These results indicate that the absence of CXCL10 activity does not ablate the capability of CD8^+^ cytotoxic T cells to migrate into the brain and eliminate *T. gondii* cysts from the brains of chronically infected mice. These results also suggest that the immune system utilizes distinct chemokines to control *T. gondii* depending on the two different life cycle stages, tachyzoite and cyst, of this protozoan parasite.

## 1. Introduction

*Toxoplasma gondii* is an obligate intracellular protozoan parasite that can infect various mammals including humans. Following the proliferation of tachyzoites (the acute stage form) during the early stage of infection, this pathogen forms tissue cysts preferentially in the brain of the hosts and establishes a chronic infection. This chronic infection is widespread in humans not only in developing countries but also in developed countries, with 30% of the population worldwide being estimated to be infected [1]. An important issue of this chronic infection in public health is an occurrence of reactivation of this persistent infection, which causes serious and potentially life-threatening toxoplasmic encephalitis (TE) in immunocompromised individuals including those with AIDS, neoplasms, and organ transplants [1,2,3,4,5,6]. TE is the most common opportunistic infection in the brain of AIDS patients [1,2,3,4,5,6]. To prevent TE, we need to develop a method to eradicate the tissue cysts of *T. gondii* from chronically infected individuals. However, there is currently no drug available to target the cyst stage of *T. gondii.*

In regard to the elimination of *T. gondii* cysts from a chronically infected host, we recently discovered that the immune system has the capability to remove the tissue cysts from the brains of infected mice through the perforin-mediated cytotoxic activity of CD8^+^ T cells [7,8]. *T. gondii* forms cysts in neurons [9,10,11] and, to a lesser extent, in astrocytes [12,13] in the brain. The formation of the cysts in neurons and astrocytes is also observed in primary brain cell cultures [14,15,16]. Therefore, the CD8^+^ cytotoxic T cells that infiltrate into the brain need to migrate to the cyst-harboring neurons and astrocytes to eliminate the cysts. Chemokines mediate the T-cell migration to their target, and previous studies by us [17] and others [18] identified that most of the CD8^+^ T cells that infiltrate the brains of *T. gondii*-infected mice express the CXCR3 chemokine receptor on their surface. There are three chemokines, CXCL9, CXCL10, and CXCL11, which bind to CXCR3. *T. gondii* infection upregulates cerebral expression of all of these three chemokines through IFN-γ [19,20]. Although our previous study showed the importance of CXCL9 for T-cell recruitment to prevent tachyzoite growth during the reactivation of cerebral *T. gondii* infection [17], CXCL9 is mostly expressed by microglia in the brains of infected mice [21]. In contrast, astrocytes express CXCL10 in the brains of mice chronically infected with *T. gondii* [21,22]. Although the chemokines expressed by neurons during *T. gondii* infection remain unknown, neuronal production of CXCL10 has been demonstrated in various microbial infections [23,24,25,26] including cerebral infection with *Plasmodium berghei* that belongs to the Apicomplexa phylum—as does *T. gondii* [26]. In addition, during the acute acquired stage of *T. gondii* infection, CXCL10 plays important roles in recruiting T cells into the livers and lungs and restricting tachyzoite proliferation [27]. Therefore, it would be possible that CXCL10 is involved in recruiting anti-cyst CD8^+^ T cells to astrocytes and neurons harboring *T. gondii* cysts for their elimination.

In the present study, we examined the role of CXCL10 in the CD8^+^ T cell-mediated elimination of *T. gondii* cysts by employing the following two approaches: (1) applying treatment with an anti-CXCL10 neutralizing monoclonal antibody (mAb) in combination with the adoptive transfer of CD8^+^ immune T cells from chronically infected wild-type mice to infected T cell-deficient SCID mice, and (2) applying adoptive transfer of the CD8^+^ immune T cells to RAG1-knockout (RAG1^−/−^) and RAG1, CXCL10-double knockout (RAG1^−/−^CXCL10^−/−^) mice chronically infected with the parasite. We found that the absence of CXCL10 activity did not affect CD8^+^ T-cell migration into the brain and elimination of cerebral *T. gondii* cysts by the T cells consistently in either of those two experimental models.

## 2. Materials and Methods

### 2.1. Mice

Wild-type (WT) BALB/c, and BALB/c-background SCID, RAG1^−/−^, and CXCL10-knockout (CXCL10^−/−^) mice were obtained from the Jackson Laboratory (Bar Harbor, ME, USA). RAG1^−/−^CXCL10^−/−^ mice were generated by mating between RAG1^−/−^ and CXCL10^−/−^ mice and maintained in our animal facility. Outbred Swiss Webster mice were obtained from Taconic (Germantown, NY, USA). Females were used in all studies. The studies were performed in accordance with the approved protocol (protocol #2020-3648) from the Institutional Animal Care and Use Committee of the University of Kentucky. There were 4 mice in each experimental group in each experiment. In the studies using SCID mice, there were 4 experimental groups in one independent replicate, and there were 3 experimental groups in another independent replicate. In the study using RAG1^−/−^ and RAG1^−/−^CXCL10^−/−^ mice, there were 4 experimental groups. In total, 54 mice were used in these studies.

### 2.2. Infection with T. gondii

SCID, RAG1^−/−^, RAG1^−/−^CXCL10^−/−^, and WT BALB/mice were infected orally with 10 cysts of the ME49 strain of *T. gondii* by gavage [7,28]. The cysts were obtained from the brains of chronically infected Swiss-Webster mice [7,28]. The T cell-deficient SCID, RAG1^−/−^, and RAG1^−/−^CXCL10^−/−^ mice were treated with sulfadiazine (Fluka, Charlotte, NC, USA) in drinking water (400 mg/L) beginning at 7 days after infection for the entire period of the experiments to maintain chronic infection in their brains [17,29]. Tissue samples of infected mice were obtained after their euthanasia with CO_2_ narcosis, followed by cervical dislocation.

### 2.3. Purification of CD8^+^ T Cells from Chronically Infected WT Mice and a Transfer of Those T Cells to Infected Immunodeficient Mice Lacking T Cells

Spleens were obtained from chronically infected WT mice, and CD8^+^ T cells were purified from their spleens using magnetic beads conjugated with anti-CD8α mAb (clone 53-6.7) (Miltenyi Biotech, Auburn, CA, USA) using the MACS system (Miltenyi) [29,30]. The purified CD8^+^ T cells were suspended in Hank’s balanced salt solution (HyClone [Cytiva], Mariborough, MA, USA) containing 2% heat-inactivated fetal bovine serum (Millipore-Sigma, Burlington, MA, USA). Infected, sulfadiazine-treated SCID, RAG1^−/−^, and RAG1^−/−^CXCL10^−/−^ mice were injected with the purified CD8^+^ T cells (2 × 10^6^ cells/0.2 mL) intravenously from a tail vein at 3 weeks after infection. Seven days later, their brains were obtained and cut into halves for freezing with dry ice for RNA purification.

### 2.4. Treatment with Anti-CXCL10 mAb

Infected and sulfadiazine-treated SCID mice were injected intraperitoneally with 100 μg of anti-CXCL10 neutralizing mAb (clone 1F11, BioXcell, Lebanon, NH, USA) every other day beginning at one day before the transfer of CD8^+^ immune T cells. As a control, another group of the infected SCID mice were injected with isotype control Ab (BioXcell) in the same manner.

### 2.5. RNA Purification and RT-PCR

RNA was purified from half of the brain of each mouse using RNA STAT-60 (Tel-test, Friendswood, TX, USA) and treated with DNase I (Invitrogen, Waltham, MA, USA) to remove genomic DNA contamination as described previously [7,31]. cDNA was synthesized from 1 or 4 μg of the DNase I-treated RNA from each brain sample. Quantitative PCR reactions were performed with the cDNA using StepOnePlus real-time PCR system (Applied Biosystems, Branchburg, NJ, USA) with Taqman reagents (Applied Biosystems) [29,30]. The primers and probes for mouse β-actin (a house-keeping control molecule), CD8β, perforin, and granzyme B (GzmB) were ready-made products from Applied Biosystems. The primers and probe for bradyzoite (cyst)-specific BAG1 are as follows: 5′-TCACGTGGAGACCCAGAGT-3′ (forward), 5′-CTGGCAAGTCAGCCAAAATAATCAT-3′ (reverse), and 5′-TTTGCTGTCGAACTCC-3′ (probe) [31]. The amounts of mRNA levels for the targets of interest were normalized to the amounts of mRNA for β-actin. In some studies, relative ratios of mRNA levels for perforin and GzmB to mRNA levels for CD8β mRNA were also calculated.

### 2.6. Statistical Analyses

The levels of significance in differences between experimental groups were determined by Student’s *t* test or Mann–Whitney *U* test using GraphPad Prism software 9.0. The latter was used when the variations in data within each experimental group significantly differed between the two groups compared. Differences that had *p* values < 0.05 were considered significant.

## 3. Results

### 3.1. Neutralization of CXCL10 Did Not Affect the Elimination of T. gondii Cysts from Infected SCID Mice After Adoptive Transfer of CD8^+^ Immune T Cells from Chronically Infected WT Mice

**Figure 1:**
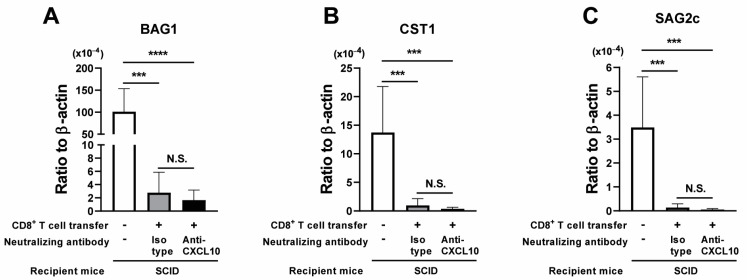
Neutralization of CXCL10 does not inhibit the effector function of CD8^+^ immune T cells to eliminate *T. gondii* cysts from the brains of chronically infected SCID mice following the adoptive transfer of T cells. SCID mice were infected with 10 cysts and treated with sulfadiazine beginning at 7 days after infection to control tachyzoite proliferation and maintain chronic infection in their brains. CD8^+^ T cells purified from the spleens of chronically infected WT mice were injected (2 × 10^6^ cells/mouse) intravenously into two groups of chronically infected SCID mice at 3 weeks after infection. One of those two groups of infected SCID mice was injected intraperitoneally with anti-CXCL10 mAb (100 μg) every other day beginning at one day before the CD8^+^ T-cell transfer. Another group was injected with isotype control Ab in the same manner. As a negative control, one group of the infected SCID mice did not receive any T cells or any Ab treatment. Their brains were obtained at 7 days after the T-cell transfer. Cerebral mRNA levels for (**A**) bradyzoite (cyst)-specific BAG1, (**B**) cyst-specific CST-1, and (**C**) bradyzoite-specific SAG2C were measured by RT-PCR. There were 4 mice in each experimental group. The date from two independent experiments were combined. *** *p* < 0.001, and **** *p* < 0.0001 by Student’s *t* test or Mann–Whitney *U* test. The latter was used when the variations in data within the experimental group significantly differed between the two groups compared. N.S.: Not significant.

SCID mice were infected and treated with sulfadiazine beginning at 7 days after infection to control tachyzoite proliferation and to maintain a latent chronic infection in their brains. At 3 weeks after infection, they received a systemic transfer of CD8^+^ immune T cells (2 × 10^6^ cells) from chronically infected WT mice in combination with intraperitoneal injections with either anti-CXCL10 mAb (100 μg) or isotype control Ab every other day beginning at one day before the T-cell transfer. As a negative control, one group of the infected SCID mice received neither any T cells nor any Abs. At 7 days after the CD8^+^ T-cell transfer (Day 7), the bradyzoite (cyst)-specific BAG1 mRNA levels in the brains of the SCID mice that had received the CD8^+^ T cells were more than 40 times less than those of the brains of the control mice that had not received any T cells, regardless of treatment with anti-CXCL10 or isotype control mAbs (Figure 1A, *p* < 0.0001 for anti-CXCL10 mAb-treated group, and *p* < 0.001 for isotype control Ab-treated group). In addition, there were no differences in the cerebral BAG1 mRNA levels between the two groups of the T-cell recipients treated with either anti-CXCL10 or isotype mAbs (Figure 1A).

Consistently, the mRNA levels for the cyst-specific CST1 and bradyzoite-specific SAG2C were both markedly lower in the brains of the recipients of the T cells regardless of the treatment with anti-CXCL10 or isotype mAbs than those mRNA levels in the brains of the control mice without the T-cell transfer (Figure 1B,C, *p* < 0.001 for both CST-1 and SAG2C). In one of two independent experiments performed, cerebral BAG1 mRNA levels in the control group with no T-cell transfer were measured at both the day of the T-cell transfer (Day 0) and Day 7. Their cerebral BAG1 did not differ between Day 0 and Day 7 (ratios of BAG1 mRNA levels to β-actin mRNA levels: 95.95 ± 39.86 [×10^−4^] at Day 0, and 96.01 ± 10.07 [×10^−4^] at Day 7]), indicating that the cerebral cyst burdens were stable between these two time points in the absence of T cells. Thus, the markedly low mRNA levels for cyst (bradyzoite)-specific BAG1, CST-1, and SAG2C in the CD8^+^ T-cell recipient groups are due to elimination of the pre-existing cysts by the T cells transferred. These results together indicate that blocking of CXCL10 chemokine activity by the neutralizing mAb does not affect the capability of the CD8^+^ T cells to eliminate *T. gondii* cysts from the brains of infected SCID mice following adoptive transfer of these T cells.

### 3.2. Neutralization of CXCL10 Did Not Affect the Recruitment of Adoptively Transferred CD8^+^ Immune T Cells into the Brains of SCID Mice Chronically Infected with T. gondii

**Figure 2:**
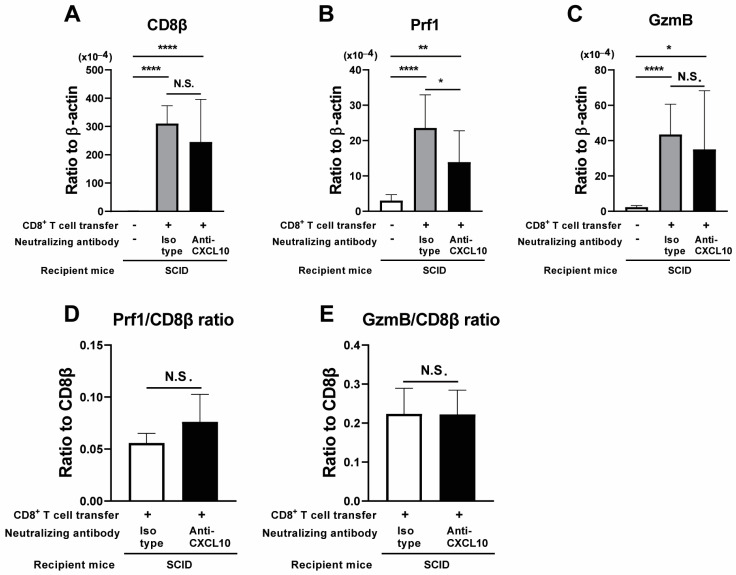
Neutralization of CXCL10 does not inhibit recruitment of CD8^+^ immune T cells into the brain and their cytotoxic activity in this organ following the adoptive transfer of the T cells into *T. gondii*-infected SCID mice. SCID mice were infected with 10 cysts and treated with sulfadiazine beginning at 7 days after infection to control tachyzoite proliferation and maintain chronic infection in their brains. CD8^+^ T cells purified from the spleens of chronically infected WT mice were injected (2 × 10^6^ cells/mouse) intravenously into two groups of chronically infected SCID mice at 3 weeks after infection. One of those two groups of infected SCID mice was injected intraperitoneally with anti-CXCL10 mAb (100 μg) every other day beginning at one day before the CD8^+^ T-cell transfer. Another group was injected with isotype control Ab in the same manner. As a negative control, one group of the infected SCID mice did not receive any T cells or any Ab treatment. Their brains were obtained at 7 days after the T-cell transfer. Cerebral mRNA levels for (**A**) CD8β, (**B**) perforin, and (**C**) GzmB were measured by RT-PCR. Relative ratios of (**D**) perforin mRNA levels to CD8β mRNA levels and (**E**) GzmB mRNA levels to CD8β mRNA levels were also calculated. There were 4 mice in each experimental group. The date from two independent experiments were combined. * *p* < 0.05, ** *p* < 0.01, and **** *p* < 0.0001 by Student’s *t* test or Mann–Whitney *U* test. The latter was used when the variations in data within the experimental group significantly differed between the two groups compared. N.S.: Not significant.

To address the efficiency of migration of adoptively transferred CD8^+^ immune T cells into the brains of the recipient SCID mice treated with anti-CXCL10 mAb, we compared the mRNA levels for CD8β in the brains of the infected, T cell-recipient SCID mice treated with anti-CXCL10 or isotype control mAbs. The cerebral CD8β mRNA levels in those two groups of CD8^+^ T-cell recipients were more than 100 times greater regardless of treatment with anti-CXCL10 or isotype control mAbs than in the control mice with no T-cell transfer (Figure 2A, *p* < 0.0001). In addition, these mRNA levels in the two groups of the T-cell recipients were equivalent each other (Figure 2A).

Since the anti-cyst effector function of CD8^+^ T cells is mediated by their perforin-mediated cytotoxic activity, we also measured mRNA levels for two key mediators of their cytotoxic activity, perforin and GzmB, in the brains of those infected SCID mice that had received CD8^+^ immune T cells. The cerebral mRNA levels for perforin and GzmB in the CD8^+^ T-cell recipients were both markedly greater regardless of treatment with anti-CXCL10 or isotype control mAbs than in the control SCID mice with no T-cell transfer (perforin mRNA levels: *p* < 0.001 for anti-CXCL10 mAb-treated group and *p* < 0.001 for isotype control Ab-treated group [Figure 2B]; GzmB mRNA levels: *p* < 0.05 for anti-CXCL10 mAB-treated group and *p* < 0.0001 for isotype control Ab-treated group [Figure 2C]). When the mRNA levels for these cytotoxic activity mediators were compared between these two CD8^+^ T cell-recipient groups treated with anti-CXCL10 mAb or isotype control Ab, the perforin mRNA levels were somehow significantly lower in the former than the latter (*p* < 0.05, Figure 2B), whereas the GzmB mRNA levels did not differ between these two groups (Figure 2C). To further address the expression of perforin and GzmB by CD8^+^ T cells that had migrated into the brains of the recipient SCID mice, we also compared the relative ratios of the mRNA levels for perforin and GzmB to the mRNA levels for CD8β in the two groups of the T cell-recipient SCID mice treated with anti-CXCL10 or isotype control mAbs. The ratios of perforin mRNA levels to CD8β mRNA levels and the ratios of GzmB mRNA levels to CD8β mRNA levels were both similarly high in these two groups of CD8^+^ T-cell recipients, and those ratios did not differ between these two groups (Figure 2D,E). These results together indicate that the blocking of CXCL10 activity with a neutralizing mAb against this chemokine did not inhibit the recruitment of CD8^+^ immune T cells into the brains of infected SCID mice that had received an adoptive transfer of those T cells, nor the expressions of the cytotoxic mediators of those T cells recruited into the brains during the elimination of *T. gondii* cysts by those CD8^+^ T cells.

### 3.3. Treatment with Anti-CXCL10 Neutralizing mAb Did Not Induce Upregulation of Cerebral CXCL10 mRNA Levels to Compensate the Blocking of CXCL10 Activity in T. gondii-Infected SCID Mice

**Figure 3:**
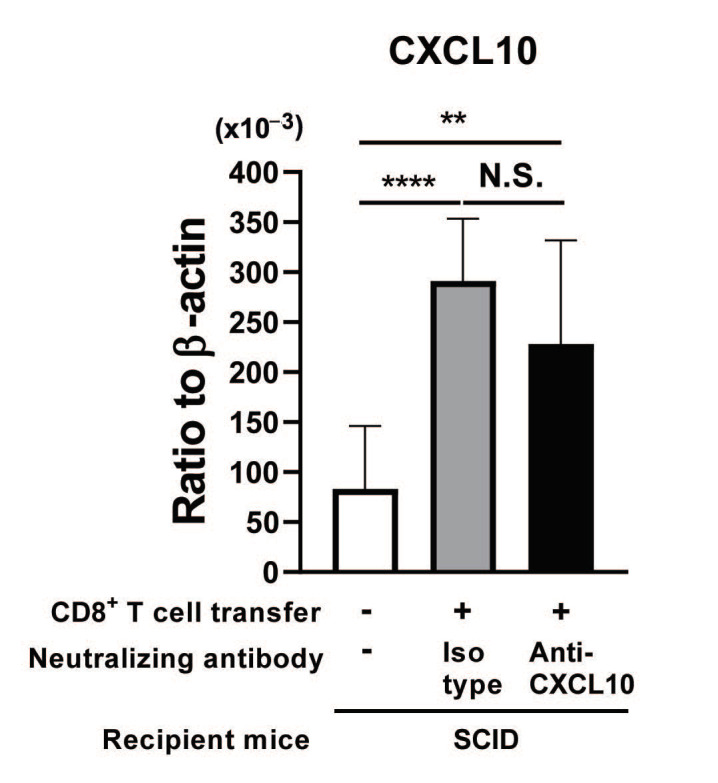
Blocking of CXCL10 activity by a neutralizing mAb does not increase the mRNA levels for this chemokine to compensate for the neutralization of the activity of this chemokine in the brains of *T. gondii*-infected SCID mice following adoptive transfer of CD8^+^ immune T cells. SCID mice were infected with 10 cysts and treated with sulfadiazine beginning at 7 days after infection to control tachyzoite proliferation and maintain chronic infection in their brains. CD8^+^ T cells purified from the spleens of chronically infected WT mice were injected (2 × 10^6^ cells/mouse) intravenously into two groups of chronically infected SCID mice at 3 weeks after infection. One of these two groups of infected SCID mice was injected intraperitoneally with anti-CXCL10 mAb (100 μg) every other day beginning at one day before the CD8^+^ T-cell transfer. Another group of infected SCID mice was injected with isotype control Ab in the same manner. As a negative control, one group of infected SCID mice did not receive any T cells or any Ab treatment. Their brains were obtained at 7 days after the T-cell transfer. The cerebral mRNA levels for CXCL10 were measured by RT-PCR. There were 4 mice in each experimental group. The date from two independent experiments were combined. ** *p* < 0.01, and **** *p* < 0.0001 by Student’s *t* test or Mann–Whitney *U* test. The latter was used when the variations in data within the experimental group significantly differed between the two groups compared. N.S.: Not significant.

A possible reason for the absence of effects of the treatment with anti-CXCL10 mAb on CD8^+^ T-cell migration into the brain and the elimination of cerebral *T. gondii* cysts by the CD8^+^ T cells in the T cell-recipient SCID mice could be that the neutralization of CXCL10 by the mAb induces an upregulation of CXCL10 production in the brains of the recipient SCID mice to maintain the effector capability of the immune system. To address this possibility, we examined whether the treatment with anti-CXCL10 mAb induced an upregulation of CXCL10 mRNA levels in the brains of the infected SCID mice. The cerebral mRNA levels for CXCL10 in the infected SCID mice that had been treated with anti-CXCL10 mAb and had received CD8^+^ immune T cells did not differ from those in the T cell-recipient mice treated with isotype control mAb (Figure 3), suggesting that the neutralization of CXCL10 activity by the mAb did not cause an increased production of CXCL10 to compensate for the loss of the activity of this chemokine by the antibody treatment.

### 3.4. The Absence of the CXCL10 Gene in T Cell-Deficient RAG1^−/−^ Mice Infected with T. gondii Rather Enhanced the Elimination of Cerebral Tissue Cysts of the Parasite Following Adoptive Transfer of CD8^+^ Immune T Cells

**Figure 4:**
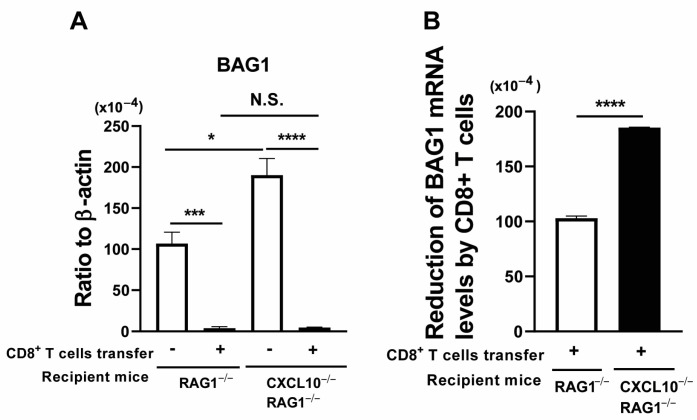
The absence of the CXCL10 gene in T cell-deficient RAG1^−/−^ mice infected with *T. gondii* rather enhances the elimination of cerebral tissue cysts of the parasite after receiving an adoptive transfer of CD8^+^ immune T cells from chronically infected WT mice. RAG1^−/−^ and RAG1^−/−^CXCL10^−/−^ mice were infected with 10 cysts and treated with sulfadiazine beginning at 7 days after infection to control tachyzoite proliferation and maintain chronic infection in their brains. CD8^+^ T cells purified from the spleens of chronically infected WT mice were injected (2 × 10^6^ cells/mouse) intravenously into one group of each of the chronically infected RAG1^−/−^ and RAG1^−/−^CXCL10^−/−^ mice at 3 weeks after infection. Another group of each of these two strains of infected mice did not receive the T cells as a control. Their brains were obtained at 7 days after the T-cell transfer. (**A**) Cerebral mRNA levels for the cyst (bradyzoite)-specific BAG1 were measured by RT-PCR. (**B**) Decreases in BAG1 mRNA levels by the transfer of CD8^+^ immune T cells were measured by calculating differences in the mean value of BAG1 mRNA levels in the control mice with no T-cell transfer and the BAG1 mRNA level of the mice that had received CD8^+^ T cells in each of RAG1^−/−^ and RAG1^−/−^CXCL10^−/−^ groups. There were 4 mice in each experimental group. * *p* < 0.05, *** *p* < 0.001, and **** *p* < 0.0001 by Student’s *t* test or Mann–Whitney *U* test. The latter was used when the variations in data within the experimental group significantly differed between the two groups compared. N.S.: Not significant.

To further confirm the results from the studies using treatment with the anti-CXCL10 mAb, we performed the adoptive transfer of CD8^+^ immune T cells from chronically infected WT mice to chronically infected (infected, sulfadiazine-treated) RAG1^−/−^ and RAG1^−/−^CXCL10^−/−^ mice. As a control, one group of each of the infected RAG1^−/−^ and RAG1^−/−^CXCL10^−/−^ mice did not receive any T cells. In the control groups without any T-cell transfer, the BAG1 mRNA levels in the brains of the RAG1^−/−^CXCL10^−/−^ mice were 70% greater than those of the RAG1^−/−^ mice (Figure 4A, *p* < 0.05). However, at 7 days after receiving the CD8^+^ T cells (2 × 10^6^ cells), the cerebral BAG1 mRNA levels of the T-cell recipients were dramatically lower than those of the control mice with no T-cell transfer in both RAG1^−/−^ and RAG1^−/−^CXCL10^−/−^ mice (Figure 4A, *p* < 0.001 for RAG1^−/−^ mice, and *p* < 0.0001 for RAG1^−/−^CXCL10^−/−^ mice). In addition, the BAG1 mRNA levels of the T-cell recipients did not differ between the RAG1^−/−^ and RAG1^−/−^CXCL10^−/−^ mice (ratios of BAG1 mRNA levels to β-actin mRNA levels: 3.86 ± 1.94 [×10^−3^] in RAG1^−/−^ mice and 4.37 ± 0.39 [×10^−3^] in RAG1^−/−^CXCL10^−/−^ mice) (Figure 4A). Notably, when the levels of reduction in BAG1 mRNA induced by CD8^+^ T cells (differences in BAG1 mRNA levels between the mice with and without the T-cell transfer) were compared between these two strains of mice, markedly greater amounts of reduction in cerebral BAG1 mRNA were detected in the T-cell recipients of RAG1^−/−^CXCL10^−/−^ than in the T-cell recipients of RAG1^−/−^ mice (Figure 4B, *p* < 0.0001).

### 3.5. The Absence of the CXCL10 Gene in T Cell-Deficient RAG1^−/−^ Mice Infected with T. gondii Did Not Affect the Recruitment of Adoptively Transferred CD8^+^ Immune T Cells into Their Brains but Enhanced the GzmB mRNA Expression in Their Brains

**Figure 5:**
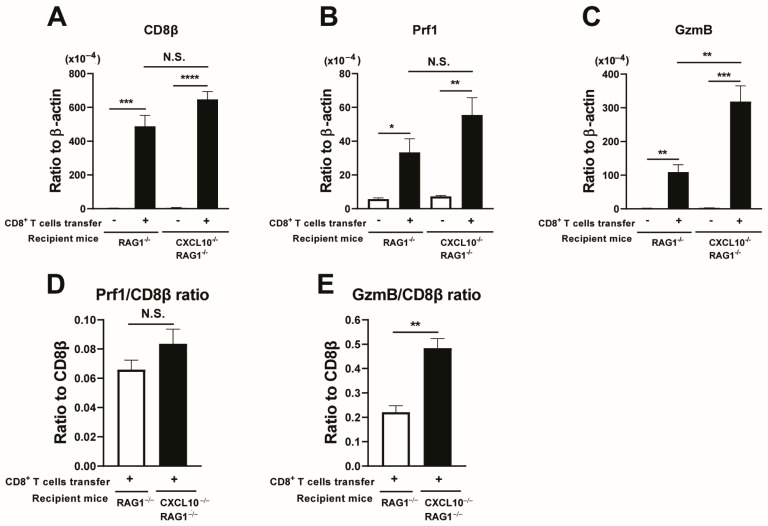
The absence of the CXCL10 gene in T cell-deficient RAG1^−/−^ mice infected with *T. gondii* does not affect the recruitment of adoptively transferred CD8^+^ immune T cells into the brain but enhances the GzmB mRNA expression in their brains. RAG1^−/−^ and RAG1^−/−^CXCL10^−/−^ mice were infected with 10 cysts and treated with sulfadiazine beginning at 7 days after infection to control tachyzoite proliferation and maintain chronic infection in their brains. CD8^+^ T cells purified from the spleens of chronically infected WT mice were injected (2 × 10^6^ cells/mouse) intravenously into one group of each of the chronically infected RAG1^−/−^ and RAG1^−/−^CXCL10^−/−^ mice at 3 weeks after infection. Another group of each of these two strains of infected mice did not receive the T cells as a control. Their brains were obtained at 7 days after the T-cell transfer. Cerebral mRNA levels for (**A**) CD8β, (**B**) perforin, and (**C**) GzmB were measured by RT-PCR. Relative ratios of (**D**) perforin mRNA levels to CD8β mRNA levels and (**E**) GzmB mRNA levels to CD8β mRNA levels were also calculated. There were 4 mice in each experimental group. * *p* < 0.05, ** *p* < 0.01, *** *p* < 0.001, and **** *p* < 0.0001 by Student’s *t* test or Mann–Whitney *U* test. The latter was used when the variations in data within the experimental group significantly differed between the two groups compared. N.S.: Not significant.

We compared mRNA levels for CD8β in the brains of RAG1^−/−^ and RAG1^−/−^CXCL10^−/−^ mice that had and had not received CD8^+^ immune T cells. Markedly increased levels of CD8β mRNA were detected in the brains of both RAG1^−/−^ and RAG1^−/−^CXCL10^−/−^ mice that had received a transfer of CD8^+^ immune T cells when compared to their control groups with no T-cell transfer (*p* < 0.001 for RAG1^−/−^ mice, and *p* < 0.0001 for RAG1^−/−^CXCL10^−/−^ mice, Figure 5A). In addition, the increased cerebral CD8β mRNA levels in the T-cell recipients did not differ between these two strains of mice (Figure 5A). Similarly, mRNA levels for perforin and GzmB were markedly greater in the CD8^+^ T-cell recipients than the control mice without the T-cell transfer in both RAG1^−/−^ and RAG1^−/−^CXCL10^−/−^ mice (perforin: *p* < 0.05 for RAG1^−/−^ mice and *p* < 0.01 for RAG1^−/−^CXCL10^−/−^ mice, [Figure 5B]; and GzmB: *p* < 0.01 for RAG1^−/−^ mice and *p* < 0.001 for RAG1^−/−^CXCL10^−/−^ mice [Figure 5C]). Notably, when comparing mRNA levels for these two mediators of the cytotoxic activity of CD8^+^ T cells between the T-cell recipients of these two strains of mice, GzmB mRNA levels were significantly greater in the brains of RAG1^−/−^CXCL10^−/−^ mice than RAG1^−/−^ mice (*p* < 0.01, Figure 5C). This result is consistent with the greater levels of reduction in BAG1 mRNA levels by a transfer of CD8^+^ T cells in the former than the latter, shown in Figure 4B. The cerebral mRNA levels for perforin were also 60% greater in the T-cell recipients of RAG1^−/−^CXCL10^−/−^ mice when compared with those of the RAG1^−/−^ mice (Figure 5B), but the difference did not reach statistical significance.

We also compared the relative ratios of the mRNA levels for perforin and GzmB to the mRNA levels for CD8β in the CD8^+^ T cell-recipients of the RAG1^−/−^ and RAG1^−/−^CXCL10^−/−^ mice. The ratios of GzmB mRNA levels to CD8β mRNA levels were significantly greater in the RAG1^−/−^CXCL10^−/−^ than in the RAG1^−/−^ mice (*p* < 0.01, Figure 5E). The ratios of perforin mRNA levels to CD8β mRNA levels also tended to be greater in the T-cell recipients of the RAG1^−/−^CXCL10^−/−^ than in the RAG1^−/−^ mice (Figure 5D), but the difference did not reach statistical significance. The results shown in Figure 4 and Figure 5 together indicate that the absence of the CXCL10 gene in *T. gondii*-infected RAG1^−/−^ mice did not inhibit either the recruitment of CD8^+^ immune T cells into their brains or the cytotoxic effector activity of the recruited CD8^+^ T cells against *T. gondii* cysts, and rather enhanced the elimination of the cysts by the T cells in association with the increased expression of GzmB mRNA after migration into their brains.

## 4. Discussion

The present study employed two different mouse models to examine the role of the CXCL10 chemokine in the CD8^+^ T cell-mediated elimination of tissue cysts of *T. gondii* from the brains of chronically infected mice. In one model, we applied blocking of CXCL10 activity by treatment with a neutralizing mAb to this chemokine in combination with the adoptive transfer of CD8^+^ T cells from infected WT mice into chronically infected SCID mice. In another model, chronically infected RAG1^−/−^ and RAG1^−/−^CXCL10^−/−^ mice received a transfer of CD8^+^ immune T cells from WT mice. These two models consistently demonstrated that the absence of CXCL10 activity did not ablate the recruitment of CD8^+^ immune T cells into their brains or the elimination of cerebral *T. gondii* tissue cysts by the T cells. Therefore, whereas CXCL10 is highly expressed in the brains of mice infected with *T. gondii* [18,19], the absence of the activity of this chemokine does not cause a defect in CD8^+^ T-cell recruitment into the brain or the anti-cyst effector function of the T cells during chronic infection with the parasite.

In the study with infected RAG1^−/−^ and RAG1^−/−^CXCL10^−/−^ mice, the cerebral BAG1 mRNA levels in the control groups that had not received the CD8^+^ immune T cells were significantly greater in the latter than the former. These mice received sulfadiazine beginning at 7 days after infection to control tachyzoite proliferation and maintain a latent chronic infection in their brains. Therefore, it is likely that tachyzoites proliferated more in RAG1^−/−^CXCL10^−/−^ than RAG1^−/−^ mice during the early stage of infection before their proliferation was controlled by sulfadiazine, and that the increased tachyzoite growth in the early stage of infection resulted in the increased cerebral cyst burden (BAG1 mRNA levels) in the RAG1^−/−^CXCL10^−/−^ mice when compared to RAG1^−/−^ mice. This possibility is supported by the evidence from a previous study [27] on significantly increased tachyzoite burdens in association with reduced T-cell numbers in the liver and lung in WT C57BL/6 mice treated with anti-CXCL10 mAb than in control mice treated with isotype mAb during the acute acquired stage of *T. gondii* infection. In contrast, the absence of CXCL10 activity did not affect CD8^+^ T-cell recruitment and the elimination of *T. gondii* cysts by the T cells from the brains of infected mice in the present study as mentioned earlier. Therefore, the roles of CXCL10 in CD8^+^ T-cell recruitment and controlling *T. gondii* most likely differ between the acute acquired stage for controlling tachyzoite proliferation and the chronic stage for eliminating the tissue cysts of the parasite.

The present study was performed using BALB/c-background T cell-deficient (SCID, RAG1^−/−^, and RAG1^−/−^CXCL10^−/−^) and WT mice to examine the roles of CXCL10 in the CD8^+^ T cell-mediated protective immunity against the cyst stage of *T. gondii*. BALB/c mice (the H-2^d^ haplotype) are suitable for this purpose, since this strain of mouse is genetically resistant to cerebral *T. gondii* infection and maintains a latent chronic infection without active tachyzoite growth in the brain [32,33,34]—as do chronically infected immunocompetent humans. In contrast, mice with the H-2^b^ haplotype, such as C57BL/6, and those with the H-2^k^ haplotype, such as CBA, are susceptible to infection, and these mice develop persistent and ultimately fatal encephalitis caused by continuous active tachyzoite proliferation in their brains [32,33,34]. A previous study by others [18] using the genetically susceptible C57BL/6 mice demonstrated that CXCL10 enhances the ability of CD8^+^ T cells to control the active proliferation of *T. gondii* in the brains during the later stage of infection. Their evidence in genetically susceptible C57BL/6 mice along with the evidence in the present study using genetically resistant BALB/c mice further supports that CXCL10 plays an important role in CD8^+^ T-cell recruitment to control the acute stage form, the tachyzoite, but not the chronic form, the cyst, of *T. gondii*.

CXCL10 is a ligand, along with CXCL9 and CXCL11, for the CXCR3 chemokine receptor. Most of the CD8^+^ T cells that migrate into the brains during the chronic stage of *T. gondii* infection express CXCR3 on their surface [17,18]. Previous studies by others [21,22] demonstrated that astrocytes are a major source of CXCL10 in the brains of genetically resistant BALB/c mice during the chronic stage of *T. gondii* infection, whereas microglia express CXCL9. The formation of cysts in astrocytes has been detected in the brains of chronically infected hosts [12,13], although neurons are more frequent host cells in the brain that harbor cysts in the later stage of *T. gondii* infection [9,10,11]. Whereas information on chemokine production by neurons in the brain during chronic *T. gondii* infection is currently lacking, the neuronal production of CXCL10 [23,24,25,26] and CXCL11 [35] has been demonstrated in various microbial infections. Therefore, it may be possible that neurons harboring *T. gondii* cysts produce both CXCL10 and CXCL11. The previous study on CXCL10 expression by astrocytes in the brains of *T. gondii*-infected BALB/c mice [21,22] did not specifically analyze the chemokine production in cyst-harboring astrocytes. Therefore, it may also be possible that cyst-harboring astrocytes express CXCL11 and/or CXCL9 in addition to CXCL10 in the brains of the chronically infected mice. If these are eventually the case in cyst-harboring neurons and astrocytes, those infected cells may be able to increase their expression of CXCL11 and/or CXCL9 in response to a functional blocking of CXCL10 or a deletion of the CXCL10 gene to maintain their capability to recruit anti-cyst CD8^+^ cytotoxic T cells to those cells for eliminating *T. gondii* cysts.

Chronic infection with *T. gondii* can reactivate and cause serious TE when the infected hosts become immunocompromised [1,2,3,4], as mentioned earlier. The reactivation of this infection is initiated by ruptures of cysts, followed by conversion of bradyzoites released from the ruptured cysts to tachyzoites, and proliferation of tachyzoites. Therefore, eradicating *T. gondii* cysts from individuals chronically infected with this parasite is an urgent need to improve public health. Since there is currently no drug effective against the cyst stage of this parasite, utilizing the capability of the immune system to target this persistent stage of *T. gondii* is an important pathway to develop a method to eradicate the cysts from chronically infected individuals and cure the infection. One third of human populations worldwide are estimated to be infected with *T. gondii* [1]. It is important to further elucidate the mechanisms by which the immune system targets and eliminates *T. gondii* cysts, including identifying chemokines produced by neurons during *T. gondii* infection, for opening a door to eradicate this widespread chronic infection in humans.

## 5. Conclusions

The present study using both the treatment of SCID mice with anti-CXCL10 mAb and a comparison between RAG1^−/−^ and RAG1^−/−^CXCL10^−/−^ mice demonstrated that the absence of CXCL10 activity does not ablate the host defense system to eliminate *T. gondii* cysts by CD8^+^ T cells from the brains of chronically infected mice, whereas the absence of CXCL10 during the acute acquired stage to control tachyzoite proliferation results in the formation of increased numbers of cysts in the brain. Our previous studies depicted that whereas the immune system utilizes IFN-γ to control the proliferation of *T. gondii* tachyzoites [36,37], it employs perforin-mediated cytotoxic activity of CD8^+^ T cells to eliminate the tissue cysts of the parasite [7,8]. This evidence along with the findings from the present study highlights the notable capability of the immune system to utilize distinct effector mechanisms to control a single pathogen, *T. gondii*, depending on the two different life cycle stages, the tachyzoite and the cyst, of the parasite.

## Data Availability

The original contributions presented in the study are included in the article, further inquiries can be directed to the corresponding author.
